# From prescription drug purchases to drug use periods – a second generation method (PRE2DUP)

**DOI:** 10.1186/s12911-015-0140-z

**Published:** 2015-03-25

**Authors:** Antti Tanskanen, Heidi Taipale, Marjaana Koponen, Anna-Maija Tolppanen, Sirpa Hartikainen, Riitta Ahonen, Jari Tiihonen

**Affiliations:** 1 Department of Clinical Neuroscience, Karolinska Institutet, Psykiatri centrum Solna R5:00 Karolinska universitets sjukhus, 17176 Stockholm, Sweden; 2 National Institute for Health and Welfare, Helsinki, Finland; 3 Department of Forensic Psychiatry, Niuvanniemi Hospital, University of Eastern Finland, Kuopio, Finland; 4 Kuopio Research Centre of Geriatric Care, University of Eastern Finland, Kuopio, Finland; 5 School of Pharmacy, University of Eastern Finland, Kuopio, Finland; 6 Research Centre for Comparative Effectiveness and Patient Safety (RECEPS), University of Eastern Finland, Kuopio, Finland

**Keywords:** Prescription register, Modeling, Drug utilization, Pharmacoepidemiology

## Abstract

**Background:**

Databases of prescription drug purchases are now widely used in pharmacoepidemiologic studies. Several methods have been used to generate drug use periods from drug purchases to investigate various aspects; e.g., to study associations between exposure and outcome. Typically, such methods have been fairly simplistic, with fixed assumptions of drug use pattern and or dose (for example, the assumed usage of 1 tablet per day). This paper describes a novel PRE2DUP method that constructs drug use periods from purchase histories, and verified by a validation based on an expert evaluation of the drug use periods generated by the method.

**Methods:**

The PRE2DUP method is a novel approach based on mathematical modelling of personal drug purchasing behaviors. The method uses a decision procedure that includes each person’s purchase history for each ATC code, processed in a chronological order. The method constructs exposure time periods and estimates the dose used during the period by considering the purchased amount in Defined Daily Doses (DDDs), which is recorded in the prescription register database. This method takes account of stockpiling of drugs, personal purchasing pattern; i.e., regularity of the purchases, and periods of hospital or nursing home care where drug use is not recorded in the prescription register. The method can be applied to a variety of drug classes with different doses and use patterns by controlling restriction parameters for each ATC class, or even each drug package. In the presented example, the PRE2DUP method was applied to a register-based MEDALZ-2005 study cohort. All drug purchases (3,793,085) recorded in the Finnish prescription register between 2002 and 2009 for persons with Alzheimer’s disease (28,093) were included.

**Results:**

Results of the expert-opinion based validation indicate that PRE2DUP method creates drug use periods with a relatively high correctness. Drugs with varying patterns of use and drugs used on a short-term basis only require more precise parameters.

**Conclusions:**

PRE2DUP method gives highly accurate drug use periods for most drug classes, especially those meant for long-term use.

## 1 Background

Starting from the 1990’s, electronic data on prescription drug purchases have been collected in the Nordic countries [1] and registers have been widely used in recent medical research [2]. These registers enable the study of relationships between drug exposure and outcomes, such as hospitalization or death. When a research question requires information on the duration of drug use, or the definition of exposure status at a specific date, methods to construct drug use periods from purchases are needed; i.e., when continuous drug use starts and ends. Currently, the durations of drug purchases have been modeled with simple assumptions, such as the use of one Defined Daily Dose (DDD) per day [3-6]. Some constants are often added to the this duration (a grace period) or the purchased amount has been multiplied with some factor to allow for lower adherence, as prescribed medications are often taken at a rate less than instructed [7]. Similarly, the assumption of one tablet per day has been used to derive estimates for specific drug use periods. Also, fixed time windows such as “purchased a drug during last 90 days before the outcome date” have been used as a proxy for current drug use [7-10]. These first generation methods may have serious weaknesses, which have been discussed by Tanskanen et al. [11]. Gislason et al. used personal, timely local dosages to estimate refill lengths based on the number of dispensed tablets [12,13]. This method is useful to identify personal variations in dosages.

This paper describes a method that represents a new approach to modelling drug exposure from register-based data of drug purchases. The PRE2DUP method is based on purchase histories and uses temporal dosages, package information with refill time distributions and patient’s personal purchase patterns. The basic idea of the method is to simulate patient’s purchase behavior and use this information to decide which purchases of a particular drug belong to the same continuous drug use period. The method has been under development since 2002, and it was first used by Tiihonen et al. in 2006 [14]. The present report describes the operation of version 14, which has been applied to drug utilization research since the spring of 2013 [15-18], and includes results from an expert opinion based validation of the drug use periods generated by the PRE2DUP method.

## 2 Methods

In the methods section, we describe the data source in sections 2.1-2.2, the functioning and calculations of the PRE2DUP method in sections 2.3-2.8 and the validation of the PRE2DUP method and statistical analyses in section 2.9.

### 2.1 Test cohort MEDALZ-2005

The MEDALZ-2005 (Medication use and Alzheimer’s disease) study included all community-dwelling persons residing in Finland with a verified Alzheimer’s disease (AD) diagnosis and who were also still alive on December 31, 2005 (N = 28,093) [19]. Patients with AD were identified from the Special Reimbursement Register. The Finnish Special Reimbursement Register contains information on reimbursement according to specific chronic diseases such as diabetes, cardiovascular diseases and Alzheimer’s disease. This data has been linked to the Prescription Register, which includes data on all reimbursed prescription drug purchases. Drugs used during hospital stays are not recorded in the register. Prescriptions purchased by the Alzheimer patients, between 1.1.2002 and 31.12.2009, were included in the study. This data has also been linked to the hospital discharge register (Hilmo) and the long-term care register. The hospital discharge register contains each person’s identification number, the date of hospital admission, diagnosis and eventual discharge of care. The long-term care register contains the person’s identification number and the starting date of care. Data from these registers is collected with personal identification numbers, but only de-identified data were used in this study. Thus, no ethics committee approval was required. The test cohort was chosen according to current research interests, and especially describes varying drug use patterns among older persons with frequent hospitalizations.

### 2.2 Information content of the prescription register

The Finnish prescription register includes purchases of all reimbursed prescription drugs within the entire country. Data in the register includes purchase date, drug substance, amount delivered in DDDs, Nordic Article number (vnr-number), package size, number of packages and strength of the drug, costs (EUR) and person identification number. Purchased drugs are coded according to the Anatomical Therapeutic Chemical –classification system (ATC) [5]. The vnr-numbers are included in the prescription register for every purchase, and these numbers were used to identify the drug package. The vnr-numbers enable the identification of each different drug package in terms of the number of tablets, strength, manufacturer and dosage form. In the prescription register, the purchased amount is also expressed in DDDs, as a measure that can easily be transformed to other units like milligrams or millilitres [5]. In Finland, drugs may be dispensed for a maximum of 3 months’ treatment per purchase.

#### 2.2.1 Pre-processing prescription register data

Since modelling is based on ATC codes and DDD values, the prescription register data was pre-processed to correct missing values in these variables. Some ATC code changes were made during the years covered in our data selection (2002–2009), and these were subsequently corrected to the current ATC code. The same method was applied to changes in DDD values. Some purchases remained without a DDD and/or a correct ATC code; for example, some reimbursed lotions that do not have any ATC code or DDD value. After these corrections, the purchases for each person and each ATC code were processed to chronological order.

### 2.3 Drug use periods, the output

The output of the method is a set of drug use periods. A drug use period is a time span describing continuous use of a certain drug (ATC code) and, thus, describes the time period when a person has used a drug. A drug use period consists of start date, end date, person id, ATC code, amount of purchased DDD, number of hospital days, number of purchases, and average DDD per day dose. Drug use period(s) are constructed for every person and for each ATC code the person has purchased. Drug use periods are timely non-overlapping for each person and each ATC code, so switching from one product to another within the same ATC code is coded as one drug use period. A person may have multiple drug use periods for the same ATC code if there is a break in purchases, so that it is not possible for a drug to have remained from one purchase to the next. A drug use period may include one or more purchases. The average dose of a drug use period is modelled as the average DDD per day, as the purchased amount is recorded as DDDs in the prescription register, and is independent of changes in units (grams, milligrams, micrograms etc.).

### 2.4 Overall functioning of prescriptions to drug use periods (PRE2DUP) method

**Figure 1:**
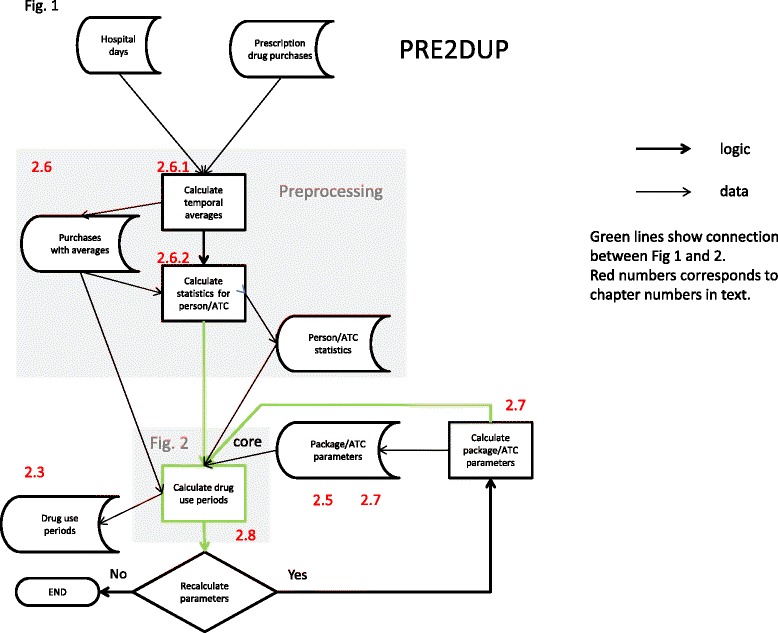
**The overall operation of PRE2DUP.** Data is first preprocessed, and then drug use periods are calculated. New parameters are calculated iteratively, to improve the results. Green arrows show the work-flow around the core process. Red numbers link to particular sections in the text.

**Figure 2:**
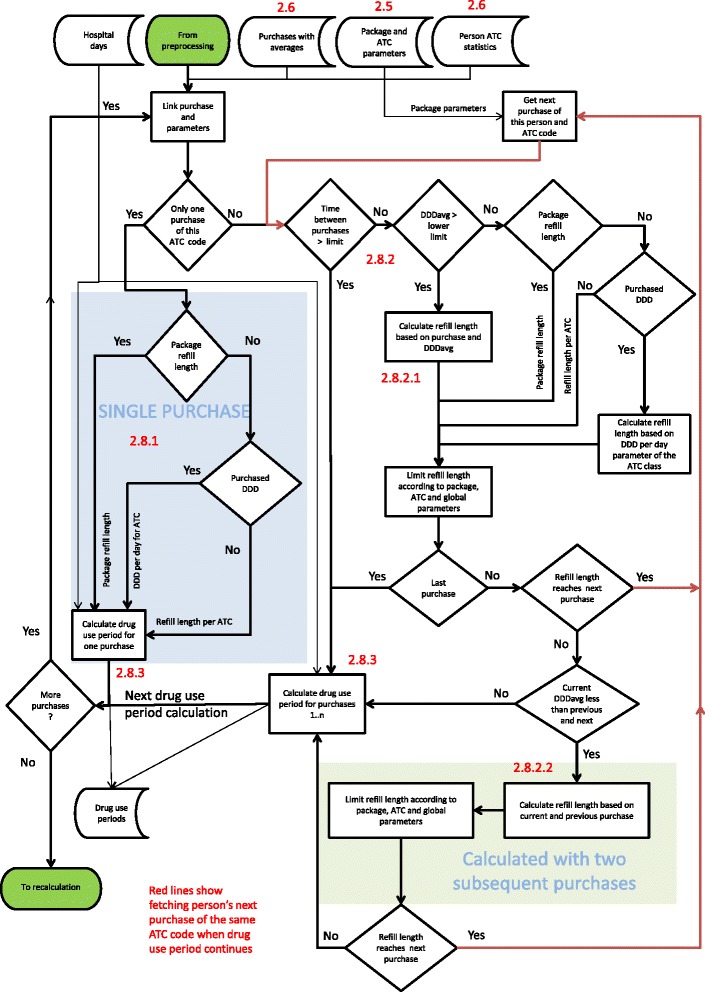
**The work-flow of the core process.** Red arrows illustrate the retrieval of a person’s next purchase of the same ATC code. The blue background marks the processing of a person’s single purchase of an ATC code. The green background shows the processing of stockpiling. Red numbers link to particular sections in the text. Thin arrows present dataflow, bold arrows logic.

PRE2DUP is based on modelling of personal drug purchasing behavior. PRE2DUP method uses a decision procedure where each person’s all purchases of one ATC code are processed in a chronological order. The method is based on temporal averages of daily dose that are used to calculate the expected refill time to next purchase. The overall system is shown in Figure 1. Initially, one defines a set of parameters for the ATC classes and drug packages (defined with vnr-numbers). Next, the preprocessing phase of the method generates temporal averages and statistics describing the regularity of each person’s purchase histories for each ATC code. After the preprocessing phase, PRE2DUP calculates the drug use periods based on drug purchases, hospital days and regularity statistics drawn from the data. The core procedure refers to calculations of drug use periods (Figure 2). Hospital days are excluded from drug use periods in this calculus. We use this exclusion for dosage calculations, not for splitting drug use periods due to short hospital stays. Exclusion of hospital stays does not imply that drug use period would stop and produce a gap to each hospitalization.

After the core has run for the first time, the method calculates refill length distribution for each vnr-number. The most common refill length is extracted from the distribution data and used when the core is run again. The most common refill length is assigned as the typical duration for a single purchase when there is no other information for the duration of drug use. This iteration process is run until the results are stable. These iterations may also include redefining the expert-defined parameter sets.

### 2.5 Expert-defined parameters

To use the PRE2DUP method, parameters for dosage limits, hospital stays and maximum refill times were formulated. These parameters restrict the joining of purchases over unrealistically long time periods (i.e., long hospital stays and drug use with doses that are too low to be realistic). Such parameters reflect, for example, reimbursement regulations and dispensing practices, which may be differ between countries. These parameters may need redefining after the first iterations of the method. The hierarchy of parameters are from specific to global, and vnr (package) parameters override ATC class parameters.

#### 2.5.1 Global parameters

Following global parameters apply to all purchases. The maximum refill time used in sliding temporal average calculations was set to 300 days. The maximum hospital stay, included as the last purchase in a drug use period, was set to 30 days. This prevents extending the drug use period over a long hospital stay. The maximum DDD per day was limited to 10, to limit possible variations when two purchases are only one or even a few days apart. Maximum length of a single purchase was set to 150 days, regardless of the purchased DDD amount. Different parameter values have been tested like 120, 150 and 180 days for single purchase or 270, 300 and 330 days for maximum refill time in sliding temporal average calculus to find a compromise for different drugs. Global parameters cannot be overridden by other parameters.

#### 2.5.2 ATC class parameters

The parameters defined for ATC classes can be defined hierarchically, starting from the main level and progressing towards finer sub-levels. The method uses for each purchase the finest level that has been defined. This is useful when some drugs in the same main ATC class are used very differently. The parameters for ATC classes include lower limits for the daily dose, the common daily dose (as DDD), the minimum length of a drug use period (days) and the maximum refill length (days) allowed in continuous use. The lower limit for daily dose guides the PRE2DUP to split drug use periods, if the calculated dose is lower than the limit. In this cohort, the cut-off was set relatively low for many ATC classes, due to the advanced age of the cohort, yet higher cut-offs could be used for different (e.g., younger) patient groups. The common daily dose is used when there is no better estimate for refill time length. We defined a total of 174 ATC parameters on different levels of the classification (Table 1).

**Table 1:** **The number of expert-defined parameters in each ATC class**

1st level ATC codes and common names	Number of ATC parameters	Number of vnr parameters
A: Alimentary tract and metabolism	31	354
B: Blood and blood forming organs	10	74
C: Cardiovascular system	13	
D: Dermatological drugs	12	
G: Genitourinary system and reproductive hormones	8	43
H: Systemic hormonal preparations, excluding reproductive hormones and insulins	6	
J: Antiinfectives for systemic use	7	
L: Antineoplastic and immunomodulating agents	5	
M: Musculoskeletal system	12	519
N: Nervous system	15	989
P: Antiparasitic products, insecticides and repellents	5	
R: Respiratory system	13	
S: Sensory organs	15	
V: Various ATC structures	22	
Total	174	2226

#### 2.5.3 Vnr-parameters

The idea of using vnr-parameters is to define upper and lower limits for the refill length of each drug package (identified by vnr-number), and these values represent the finest level of expert-defined parameters. These values are based on the pharmaceutical properties of the drug in terms of dosage form, the assumed pattern of use, longevity after opening and the number of divisible units. Vnr-parameters include minimum refill length, maximum refill length, typical refill length and corresponding DDD per day values. Maximum refill length for a package is based on the number of included units (e.g.; tablets, capsules, injections), how they dividable and how often this drug has to be taken (e.g.; once a day, once a week, etc.). For example, a package containing 30 non-dividable capsules for a drug taken once daily, the vnr-parameter for maximum refill length may be set to 30 days (or to allow some non-adherence, for example 1.2 ×30 days). Minimum refill length may be set to 15 days if 2 capsules per day represent the maximum dose per day. A typical refill length would be 30 days. We defined the vnr-parameters for 2226 packages based on the current research interests, and included mainly ATC classes, such as N (Nervous system) and M (Musculo-skeletal system) (Table 1).

### 2.6 Preprocessing phase

The preprocessing phase calculates variables that are used by the core process and do not change during iterations. Preprocessing calculates refill times between each person’s purchases of one ATC code, the number of hospital days between purchases, temporal averages of daily doses as DDD per day, referred as DDDAVG, and variation of the daily dose (i.e., the coefficient of variation for DDDAVG, referred as DDDAVGcv).

#### 2.6.1 Temporal average

For each purchase, a sliding weighted temporal average for dose (DDDAVG_i_) is calculated from each persons’ subsequent purchases of the same ATC class as follows:

1 $$ DDDAV{G}_i=\frac{DD{D}_{i-1}+4DD{D}_i+DD{D}_{i+1}}{T_{i-1}+4{T}_i+{T}_{i+1}} $$

where DDD_i_ is the DDD amount that is purchased at time i, and T_i_ is the number of days between purchase i and i + 1. The consecutive drug purchases are weighted with weights of one, four and one for i-1, i and i + 1 respectively. Weights were selected to give high weight for current purchase and equal weights for previous and next purchase. For each person’s first purchase of each ATC-code, DDD amount and time weights are five for i, one for i + 1, and for the last purchase one for i-1 and five for i. For the last purchase i, T_i_ is calculated from the previous purchase while assuming the same DDD per day value as the previous purchase, T_i_ = DDD_i_/(DDD_i-1_/T_i-1_). The assumption that dose does not change is used for convenience. When the time between two purchases is over the maximum refill time (300 days), it is assumed that they do not belong in the same treatment period, and thus, the DDDAVG calculus is ended and restarted after this time gap. Average DDDs per day are only calculated when there are at least three purchases of the same ATC code for one person. When there are less than three purchases of an ATC code, such purchases are collectively transferred to the core process without pre-processing. Hospital days are excluded (i.e., days in hospital have been subtracted from T).

#### 2.6.2 Coefficient of variation for DDDAVG

The coefficient of variation for DDDAVG (DDDAVGcv) describes the regularity of a purchase pattern, and is calculated for each patient and for each ATC code, where DDDAVGcv is the standard deviation of DDDAVG divided by the mean of DDDAVG. The coefficient of variation is large if there are a lot of variations in refill times T_i_ and/or purchased DDD_i_. The coefficient of variation for DDDAVG is calculated when DDDAVG is calculated for a particular ATC code (i.e., when there are more than two purchases of the same ATC code and the purchased amount is recorded in DDDs).

### 2.7 Package refill lengths

**Figure 3:**
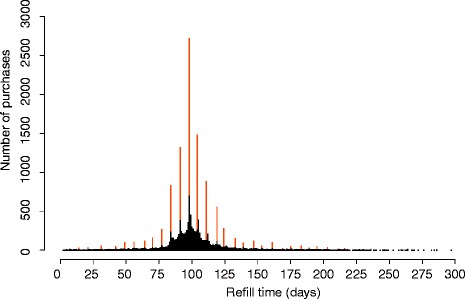
**Refill length distribution of simvastatin (vnr 010940, 10 mg, 98 tablets, ATC C10AA01).** The most common refill length (98 days) is the number of tablets in the package, which corresponds to a dosage of 0.33 DDD per day (as 1 tablet per day). Black bars are the original refill times and brownish bars joined ones.

Distributions of package refill lengths are calculated for each vnr-number, in order to define the most common refill time length in the study population. When the core of the model has run for the first time, a new parameter space is generated for package refill lengths. The distributions of refill lengths are calculated from drug use periods that include the particular package (identified by vnr-number) for each vnr-number. This is done by joining together the number of refill lengths to the nearest local maximum. From these refill length distributions we selected a mode to present the expected refill length for each package. Only modes having more than a threshold value of purchases are used, and the threshold was set to 10 purchases. We included only drug use periods that had at least six purchases to calculate the refill lengths for each package. These drug use periods may also include purchases of other packages (other vnr-numbers), but purchases including multiples of different packages are not used, as to avoid problems with multiple strength dosages. The refill lengths are used as such without subtracting hospital stays. Purchases dispensed as a dose dispensing service were not included in the calculation of refill lengths, as the whole package is not dispensed to one person in such cases. Figure 3 shows a distribution of original refill lengths (with black bars) and processed refill lengths (with brownish bars). In unprocessed refill length distributions (black bars), the peak is at 98 days (14 weeks) with 692 purchases, and after joining the refill time lengths to the nearest local maximum, the peak then includes 2718 purchases (brownish bars). Sometimes joining produces a different peak time than the highest peak in the original distribution.

### 2.8 Calculation of drug use periods, the core process

Figure 2 shows the core of the algorithm, where the decision is made to determine which consecutive purchases belongs to the same drug use period. When this core is run for the first time, it has no method-generated parameters (such as package refill lengths), and uses only expert-defined parameters (section 2.5). In the following runs, expert-defined parameters for typical refill lengths are overrun when there are enough purchases of a package in question, typically hundreds of purchases, so that refill time lengths may be calculated from the data.

The calculation of drug use periods differ for single purchases, when there is no joining procedure and no personal purchase history for the ATC code in question. When a purchase history includes more than two purchases of the same ATC code, we primarily use the person’s own purchase history, secondly package (vnr) parameters and thirdly the ATC class parameters. Even if a person has more than one purchase for certain ATC code, some purchases are treated in this process as single purchases if they are far away from each other in time. For single purchases, PRE2DUP uses information gathered from other persons’ purchases of a particular drug package (package refill lengths, see 2.7). If a person has two purchases of an ATC code, the core procedure tests to see if these two purchases are not too far apart to be joined into same drug use period, and this is done with vnr- and ATC parameters.

#### 2.8.1 Single purchase

If a patient has only one purchase of a certain ATC code, the model uses the most common refill length for the purchased package in the study population (see section 2.7). The length of drug use period is the typical refill length multiplied with the number of purchased packages. If the most common refill length is not available, due to a rarity of purchases of the package, the expert-defined typical DDD per day value for this package or ATC class is used to calculate the length of drug use period. The length is calculated as [purchased DDD(1 + DDD constant for non-perfect adherence =0.2)]/recommended DDD per day. However, the maximum length for a single purchase was limited to 150 days.

If a person has a purchase that is modelled as a separate drug use period, and some longer drug use periods of the same ATC code (for example two years apart), the DDD per day for the single purchase is adopted from the person’s nearest drug use period having at least two purchases.

If refill time length of the package in the study population and purchased amount in DDD are missing, then the ATC class-defined minimum length for drug use period is used.

#### 2.8.2 Connecting purchases

When person has more than one purchase of one ATC code, the method evaluates consecutive purchases to form drug use periods. The process utilizes data from hospital stays, purchases and data produced by the pre-processing phase of the method. The connection is made if the purchased amount is calculated to last until the next purchase.

##### 2.8.2.1 Expected refill length

The PRE2DUP algorithm connects purchases based on the local DDDAVG_i_ value for purchase i.

The expected refill length of purchase i is referred to as ERFL_i_, which is calculated as follows:

2 $$ ERF{L}_i=\frac{DD{D}_i\times \left(1+ DVAR\times DDDAVGcv\right)}{DDDAV{G}_i} $$

Where DDD_i_ is purchased amount, 1 + DVAR*DDDAVGcv is a multiplier that reflects a persons’ regularity of purchases, and DVAR is set to 0.5. Thus, 50% of the coefficient of variation is added to the multiplier. The DDDAVG_i_ value is compared to the lower limit of DDD per day that is set for the purchased package (section 2.5.3), or when no vnr-parameter is available the DDDAVG_i_ that is set to the lower limit of the ATC class (section 2.5.2).

If the DDDAVG_i_ is lower than the preset ATC lower limit, it means that the next purchase is further away than the regular drug use pattern this drug would allow, and the algorithm uses package refill length to calculate the duration of the purchase and the drug use period is ended after this purchase. If the refill length for this package is not available, the algorithm uses the common DDD per day of the ATC class when the purchased amount is recorded in DDDs. In a rare case, when a DDD value is missing, the algorithm uses expert-defined refill length for this ATC class. ERFL_i_ is thus limited according to the package and ATC parameters, in order to avoid unrealistically long refill lengths. This is done to limit ERFL_i_ in cases of large stockpiling, irregular use of a drug (take “when needed”) or a restart of drug use after a long break. This limiting process truncates drug use period according to common continuous use pattern.

If the DDDAVG_i_ exceeds a preset upper limit for daily dose, we use the limit instead of DDDAVG_i_ for determining the length of the purchase period. This may happen when purchases are only one or a few days apart.

If the current purchase happens to be the last one for an ATC code the drug use period ends and algorithm starts to process the person’s purchases of the next ATC code. If there are more purchases of this particular ATC code, the algorithm tests whether or not the calculated refill length for this purchase reaches the next purchase. If it reaches the next purchase, the algorithm starts to process the next purchase. If a calculated refill length is shorter than the time until the next purchase, the method tests whether or not there has been stockpiling before the current purchase.

##### 2.8.2.2 Stockpiling test

The stockpiling test compares DDDAVG_i_ with the DDDAVG of the previous purchase (DDDAVG_i-1_) and the following purchase (DDDAVG_i+1_). The rationale for this test is that changes in the sliding temporal average over three subsequent purchases is insufficient to take stockpiling into account. A simple example, presented in Table 2, describes the function of this test. In this example, the second purchase represents possible stockpiling (i.e., short intervals between purchases) and the third purchase represents the use of this stock (i.e., long intervals between purchases). The DDDAVG_3_ is 0.8 and this gives a calculated refill time of 12.5 days. Here, the DDDAVG_3_ is lower than DDDAVG_2_ and DDDAVG_4_. Thus, the drug use period continues over this interval.

**Table 2:** **An example of purchase history, consisting of five purchases; each of 10 DDDs and average dose is one DDD per day**

Purchase i	1	2	3	4	5
DDD	10	10	10	10	10
Days between i and i + 1	10	5	15	10	NA
DDD per day to next purchase	1,00	2,00	0,67	1,00	NA
Sliding temporal average DDDAVG_i_	1,09	1,33	0,80	0,92	1,00
Calculated refill time length in days	9,17	7,50	12,50	10,83	10,00

The second purchase shows a possible example of stockpiling and the third purchase suggests the use of this stock.

If the DDDAVG_i_ is not lower than the previous DDDAVG_i-1_, or the following DDDAVG_i+1_, then the drug use period ends and is entered into the drug use period set. If the DDDAVG_i_ is lower than the previous and the following DDDAVG values, then the algorithm calculates a refill time length with the current and previous purchases taken together, as described above for one purchase (section 2.8.2.1). This means that the refill length is calculated as two subsequent purchases, which would have happened at the time of the first purchase. If the calculated refill length for these two purchases (taken together) reaches the next purchase, then algorithm starts to process next purchase; i.e., the drug use period continues. Otherwise the current drug use period ends.

#### 2.8.3 Calculating the length of a drug use period from the last purchase

The end of a drug use period is calculated from the last purchase. When a drug use period contains more than one purchase, the algorithm uses the purchased DDD of the last purchase at date_i_, so that the DDDAVG_i_ and the number of purchases belong to this drug’s use period (denoted by k, in Equation 3):

The end date of a drug use period is calculated from the last purchase i as

3 $$ END=dat{e}_i + \frac{DD{D}_i\times \left(1+0.5\times DDDAVGcv\right)}{DDDAV{G}_i\times \left(1+{e}^{-k}\right)}+H{D}_i $$

where coefficient 1 + e^-k^ limits stockpiling for periods with very few purchases. HD_i_ is the number of hospital days that fall within the calculated time after the last purchase that is added to the drug use period. If the person is hospitalized before the drug supply of the last purchase ends, the number of hospital days is added to the length of the last purchase as long as there is no continuous hospital stay exceeding the predefined limit (in our example, 30 days).

If there is no data on purchased DDD_i_ or no DDDAVG_i_, then the duration of the last purchase is calculated as in case of single purchase, but using the last purchase instead of the first (only). This case applies only when there are two purchases of this ATC code for one person, or when the purchased amount in DDD is not registered.

### 2.9 Validation of the results produced by PRE2DUP

The validity of calculating drug use periods produced by the PRE2DUP method was assessed by two independent reviewers with expertise in clinical pharmacy (HT and MK). We did two separate validations; one with purchases (referred as the purchase test) and another with drug use periods (referred as the drug use period test). In the purchase test, we randomly selected 1000 purchases and rated them as correctly placed in a drug use period (i.e., single purchase period, included in a correct drug use period or being either the end or the beginning of a period). Purchases that lacked sufficient information for making a decision (e.g., no DDD or package information) were classified as non-solvable. The classes were correct, wrong and non-solvable.

In the drug use period test, we randomly selected 1000 drug use periods to determine if they included the correct set of purchases, or not. This means that the first purchase was correct, the subsequent purchases belonged to this drug use period and the last purchase correctly ended the period. In addition, possible purchases after the last one and before the first in the drug use period were also evaluated, to determine of if they should have belonged to the drug use period, or not. In this case, the logical options were; 1) the drug use period consisted of correct purchases, 2) the drug use period was incorrectly generated (i.e., the start or end of the purchase period was incorrect, or it should be divided into two or more periods), 3) there was not enough information to judge correctness. In the presentation of the results for both tests, we defined the “correct” option when at least one of the reviewers stated the purchase or drug use period as correct, and “error” when both reviewers agreed that it was erroneous, and the rest as “non-solvable”. To assess the reliability of the two expert opinions, we used Krippendorffs alpha (K-alpha) [20] to measure of how each reviewer’s opinions matched with each other.

The PRE2DUP method has been implemented with dBase 2.8 (dBase LLC, Binghampton, NY). Statistical analyses were performed with SPSS version 22 (IBM corp., Armonk, NY).

## 3 Results

### 3.1 Drug use periods

PRE2DUP generated 703,839 drug use periods from 3,793,085 purchases. The total number of purchased DDDs was 250 million, with a total drug use period length of 0.89 million years. The mean length of a drug use period was 458 days, and the total length was 31 years per person. This implies a high rate of polypharmacy during a seven year follow-up time. There were 9,583 purchases without an ATC code in the data. The longest mean durations were found in ATC classes C (Cardiovascular system) and L (Antineoplastic and immunomodulating agents); 864 and 778 days, respectively (Table 3). These ATC classes also had the highest DDD amounts per drug use period, with medians of 335 (ATC class C) and 300 (ATC class L). Drugs in these ATC classes are mostly for continuous use. The shortest median duration was in ATC class P (Antiparasitic products), where the drug use periods mostly had a single purchase, and thus did not include enough information to count typical refill times. In this case, the median length of drug use was 15 days, which was the preset minimum length for a drug use period. The average DDD per day was higher than the median for all ATC classes except G; which implies a skewed distribution. The group OTHER was a mixture of ATC classes that lack DDD values (e.g., ATC classes D for Dermatologicals, S for Sensory organs [mostly eye and ear drops] and V for Various).

**Table 3:** **Purchases and drug use periods by ATC class among persons with Alzheimer’s disease in the MEDALZ-2005 data during years 2002-2009**

ATC CLASS	Purchases	Drug use periods								
	N	N	Number of purchases per drug use period		DDD of drug use period		Length in days		DDD per day	
			Median	Mean	Median	Sum	Median	Mean	Median	Mean
A	402,210	82,735	2	5	100	3,2475,207	150	418	0.85	1.00
B	116,233	19,422	3	6	200	7848,540	351	638	0.64	0.87
C	1,075,095	124,811	5	9	300	8,6698,771	573	864	0.60	0.78
G	130,913	21,662	3	6	133	8886,697	241	529	0.78	0.78
H	86,325	14,297	3	6	133	5062,266	253	615	0.60	0.76
J	155,495	94,577	1	2	8	2040,569	21	106	0.33	0.38
L	17,547	2,061	6	9	335	1308,533	566	778	0.80	0.81
M	202,855	70,855	1	3	33	1,0474,142	50	197	0.75	0.77
N	1,328,357	173,702	4	8	140	8,3409,222	320	596	0.68	0.75
P	3,445	1,366	1	3	6	95,713	15	217	0.40	0.42
R	117,953	29,594	1	4	64	7443,245	138	349	0.67	0.74
OTHER	156,657	68,757	1	2	0	5085,132	28	147	0.00	0.11
TOTAL	3,793,085	703,839				250,828,037				

PRE2DUP created 703,839 drug use periods from 3,793,085 purchases. The total number of purchased DDDs was 250,828,037. OTHER included ATC classes D, S and V. The common names of ATC classes are found in Table 1.

### 3.2 Validity of drug use periods

Two independent reviewers evaluated whether or not purchases were correctly placed in drug use periods, and results of the purchase test are shown in Table 4. Both reviewers judged 87% (N = 867) of the examples as correct and 4% (N = 39) as erroneous. The proportion of purchases that at least one of the reviewers had judged as correct was 92%. The amount of non-solvable cases was 5% and 4% for the reviewers MK and HT, respectively. The reliability between reviewers opinions, as measured by Krippendorffs alpha, was 0.67 (95% Cl 0.5, 0.82). The number of disagreements between reviewers was 65 (6.5%) for all purchases.

**Table 4:** **Reviewers’ judgments on the correctness of placing a purchase in a correct drug use period (purchase test)**

Purchases		Reviewer HT			Total
		Correct	Error	Not Solvable	
Reviewer	Correct	867	17	7	891
MK	Error	16	39	2	57
	Not Solvable	14	9	29	52
	Total	897	65	38	1000

The drug use period test evaluated if the drug use periods included the correct set of purchases, or not. Both reviewers judged that 64% (N = 635) of the drug use periods were correct and 8% (N = 84) were erroneous (Table 5). The inter-reviewer Krippendorff alpha was 0.66 (95% CI 0.54, 0.78).

**Table 5:** **Reviewers’ judgments on the correctness of drug use periods (drug use period test)**

Periods		Reviewer HT			Total
		Correct	Error	Not Solvable	
Reviewer MK	Correct	635	36	10	681
	Error	82	84	2	168
	Not Solvable	23	4	124	151
Total		740	124	136	1000

The validity of the PRE2DUP methods varied between different ATC classes. The best results for the purchase test were achieved for ATC classes A, B, C, G, N and R, where the rate of correct placements was over 90% (Table 6). This was measured so that at least one of the two reviewers judged it correct. Error rates were high for ATC classes H and J and in the group described as “OTHER” (i.e.; ATC D, L, P, S and V), where there were a remarkable proportion of non-solvable cases. When comparing the performance against the number of expert-defined parameters we can see that there were no vnr-parameters for ATC class J and H and the group “OTHER” (Table 1).

**Table 6:** **Distribution of reviewer judgments by ATC classes in the purchase test, which measured correct purchases in this drug use period**

		Classification			Total N		%	
		Correct	Error	Non solvable		Correct	Error	Non solvable
ATC class	A	89	2	1	92	97	2	1
	B	44	0	0	44	100	0	0
	C	275	2	13	290	95	1	4
	G	37	1	0	38	97	3	0
	H	21	6	1	28	75	21	4
	J	28	12	0	40	70	30	0
	M	46	5	4	55	84	9	7
	N	325	8	4	337	96	2	1
	R	32	2	1	35	91	6	3
	OTHER	24	1	16	41	59	2	39
Total		921	39	40	1000	92	4	4

The category “OTHER” included classes D, L, P, S and V. These have been combined, due to the low numbers of purchases and poor data quality (high proportion of missing DDD values). The common names of ATC classes are found in Table 1.

The rate of correct drug use periods was lower in the drug use period test than in the results of the purchase test. ATC classes A, B, and N had correctness rates over 90% and G and M over 80% (Table 7). High error rates were found to be over 10% in ATC classes G, H, J and R. The amount of non-solvable drug use periods was extremely high in the group described as “OTHER” (63%), which was caused by the lack of DDDs in this data. The average performance of the method was fairly high, as 79% of all drug use periods were classified as correct and only 8% erroneous. Especially for the ATC class N, which had a high correctness rate, and this was achieved with comprehensive vnr-parameters (Table 1).

**Table 7:** **Distribution of reviewer judgments by ATC classes on the correctness of drug use periods**

		Classification			Total		%	
		Correct	Error	Not solvable		Correct	Error	Not solvable
ATC class	A	117	7	5	129	91	5	4
	B	24	2	0	26	92	8	0
	C	135	4	34	173	78	2	20
	G	32	7	0	39	82	18	0
	H	11	6	1	18	61	33	6
	J	104	43	0	147	71	29	0
	M	77	3	12	92	84	3	13
	N	217	5	2	224	97	2	1
	R	31	5	9	45	69	11	20
	OTHER	38	2	67	107	36	2	63
Total		786	84	130	1000	79	8	13

The category “OTHER” included classes D, L, P, S and V. These have been combined, due to the low numbers of purchases and poor data quality (high proportion of missing DDD values). The common names of ATC classes are found in Table 1.

## 4 Discussion

PRE2DUP is based on modelling of drug purchasing behavior, and it creates drug use periods with high precision. The results of the validation show that with a comprehensive parameter space, it can yield highly reliable solutions. The correctness of estimating the drug use periods depends on how precisely the parameters are defined. The most accurate results are gained with the parameterization of drug packages through vnr-numbers. Results show that the method calculates correct drug use periods with very few expert-defined parameters for drugs that have a regular use pattern. Drugs that tend to have a varying pattern of use (i.e., multiple indications with different dosage ranges, for example antipsychotics), and for drugs that are only used on a short-term basis (such as antibiotics), or with seasonal variation patterns that require more precise parameters.

The low value of Krippendorffs alpha in the drug use period test needs some clarifications. The rates of erroneous and non-solvable drug use periods were higher in the drug use period test than in the purchase test. This may be due to the multiple judgments that have to be made when there are multiple purchases in the evaluation of the drug use period test. Further, the distributions of ATC classes that were randomly selected for validation were different in the two validation sets, which may also affect the results. The likelihood to be randomly selected for the purchase test was high when the number of purchases per ATC class was also high. The likelihood to be randomly selected for drug use period test was high when the number of drug use periods per ATC class was high (and, subsequently low when the number of drug use periods was also low, which may indicate long and continuous drug use periods). The number of purchases included in the period affects the correctness of the drug use period, because a decision of correctness is made for every purchase in turn. Thus, it is proportional to the product of single decisions for correctness of all purchases in the drug use period. It is also important to remember that erroneous results in validation are not necessarily completely wrong, and erroneous can also mean that a drug use period was one week too long after the last purchase, or two purchases were joined over a time gap that both reviewers judged to be two weeks too long.

The PRE2DUP method is more flexible than previous methods that are mostly based on simple assumptions, such as one tablet per day or one DDD per day, and has several advantages over such first generation methods [11]. Most importantly, PRE2DUP uses personal purchase histories with no single fixed dosage assumptions, and both dosage and purchase patterns are calculated individually for each person and each drug. Thus, it produces a better estimate of an actual drug use pattern with the consideration of natural variations in usage for each person. Secondly, PRE2DUP effectively utilizes package information, both through expert-defined parameters and through parameters calculated from purchase histories (i.e., package refill lengths). This produces a set of reliable parameters that match with actual drug use patterns in a particular study population, instead of relying on manufacturers’ instructions or other assumptions. Thirdly, the PRE2DUP can be guided to more optimal results through its recursive nature, and it is possible to target fine-tuning towards problematic drug classes, or even the use of single packages. Fourthly, it can be adapted to different levels of information. For example, if there is no package information available, the sub-routines using package information are not used and the method only uses purchased DDD values and ATC class parameters.

The vast parameter space may be difficult to control, and anomalies may exist if there are very different subpopulations. To solve this problem, we treated patient cases and their controls separately in the MEDALZ-2005 study, because the cases have the diagnoses of Alzheimer’s disease, whereas controls did not. It may sometimes also be useful to split a population, for example according to age, and then process drug use separately for children, adults and older persons.

We have not dealt with the question of how long different drugs remain in body after the end of a drug use period. This is one thing that could be added to the calculus for some specific drug classes. However, there is no data on drug use after the last purchase, and purchased stock is not always used completely. Dealing with long elimination half-lives of drugs after the last purchase may require information on gender and age, which are routinely available from the registers, but possibly also the weight of the patient is required, which is rarely available.

The PRE2DUP method is evolving and new features are added according to the needs that may arise from various research questions, population characteristics or content of the registers. Our goal is to make the method as data driven as possible; i.e., minimize manual tuning and parameterization. In this effort, we have presented a method to convert drug purchases to drug use periods. We hope that it leads to the development of new methods and improvements in register-based pharmacoepidemiology.

We presented the application of PRE2DUP method to a specific population (persons with Alzheimer’s disease). The population is characterized by a high number of comorbidities, concomitant drugs and frequent hospitalizations and thus, it represents ultimate challenge to modelling of drug use. However, these characteristics are not requirements for this method. We have used earlier simpler versions of this method with persons with mental disorders and ATC N group drugs [14,21,22] and we also have applied the method for drug purchases of matched controls without Alzheimer’s disease in this data. A limitation is that we tested the method only to Finnish data and Finnish prescription and dispensing regulations. We have not compared the drug use periods produced by the PRE2DUP with other methods in this study. The strengths of this study are large prescription data with long follow-up time, and ability to model use of all different drug groups with various patterns of use.

We do not know any other studies, where large prescription dataset with all different drugs used (over 700 different ATC codes) by large population (over 50 000 persons together) has been modelled with a single method. Previous studies have concentrated on one or a couple of related drugs and methods have been tailored for these particular cases. The strength of PRE2DUP is that it treats all patients and drugs individually and possible bias in timing of drug exposure is not linked to predefined dosage (DDDs or tablets) nor to regularity of purchases (fixed time windows). This is very important when the outcomes are related to dosage or adherence and bias in calculated drug exposure is larger with atypical drug use than in common drug use.

## 5 Conclusions

Previously, drug use periods have been calculated with fixed assumptions of dosage and refill length. These methods do not take into account personal dosage, dosage changes over time or differences in purchase behavior and may lead to incorrect results. PRE2DUP method uses personal drug purchase histories and fits parameters for each person and drug. It uses package information effectively and produces highly correct estimates for drug use periods for most drug classes.

Studies comparing different ways to convert prescription data to drug use periods are needed in different settings (patient groups, drugs and countries). With these comparative studies researchers can choose the best method or develop new ones for their own settings.

## Abbreviations

ATC: Anatomical Therapeutic Chemical
DDD: Defined daily dose
